# Hypoxia-induced PD-L1 expression and modulation of muscle stem cell allograft rejection

**DOI:** 10.3389/fphar.2024.1471563

**Published:** 2024-11-01

**Authors:** Jacob Raiten, Genevieve M. Abd, Shane B. Handelsman, Harshank V. Patel, Jennifer C. Ku, Agata M. Parsons, Jonathan L. Wassink, Sheridan L. Hayes, Juliana Overbay, Yong Li

**Affiliations:** ^1^ Western Michigan University Homer Stryker M.D. School of Medicine, Kalamazoo, MI, United States; ^2^ Division of BioMedical Engineering, Department of Surgical Science, Western Michigan University Homer Stryker M.D. School of Medicine, Kalamazoo, MI, United States

**Keywords:** cell therapy, stem cell transplantation, duchenne muscular dystrophy (DMD), Programmed death ligand 1, immune rejection, hypoxia preconditioning, muscle stem cells

## Abstract

Stem cell therapy has shown immense promise in treating genetic disorders, particularly muscular diseases like Duchenne muscular dystrophy (DMD). This study investigates a novel method to enhance the viability of stem cell transplants in DMD by upregulating Programmed Death Ligand 1 (PD-L1) in muscle stem cells (MuSCs) through preconditioning with hypoxia and/or interferon-γ (IFN-γ) to mitigate T cell immune rejection. MuSCs were treated with 5% hypoxia for 72 h and further treated with IFN-γ to enhance PD-L1 expression. Additionally, gain and loss experiments using a PD-L1 inhibitor (BMS-1) were conducted to investigate cellular expression profiles *in vitro* and cell transplantation outcomes *in vivo*. Our results showed significant upregulation of PD-L1 in MuSCs under hypoxia and IFN-γ conditions without affecting cellular proliferation and differentiation *in vitro*. *In vivo*, these preconditioned MuSCs led to decreased infiltration of CD4^+^ and CD8^+^ T cells in implanted limb muscles of mouse models. Blocking PD-L1 reduced graft survival in muscles treated with MuSCs. Conversely, increased PD-L1 expression and reduced T cell infiltration correlated with improved graft survival, as identified by pre-labeled LacZ + MuSCs following transplantation. This study provides evidence that hypoxia and IFN-γ preconditioning of MuSCs can significantly enhance the efficacy of cell therapy for DMD by mitigating immune rejection. Our strategic approach aimed to improve donor cell survival and function post-transplantation by modifying immune responses towards the donor cells.

## Introduction

Stem cell therapies and cell transplantation have burgeoned following James Thomson’s 1998 discovery of embryonic stem cells (Thomson et al., 1998). Over the last three decades, the convergence of regenerative medicine, immunology, and tumor biology has ushered transformative medical interventions in conditions including leukemia, lymphoma, and genetic disorders through stem cell transplants (Candoni et al., 2019; Taylor et al., 2019; Samara and Mei, 2022; Forlanini et al., 2023). These therapies hold immense potential for regenerating damaged tissues, with one promising application being the treatment of muscular dystrophy (Sienkiewicz et al., 2015). Malfunctioning dystrophin is a primary cause of Duchenne muscular dystrophy (DMD), characterized by ischemic damage and myocyte cell death, resulting in the replacement of functional muscle tissues with fibrotic and non-functional adipose tissue (Tsuda, 2018). One proposed treatment for DMD involves replacing diseased muscle cells with stem cells carrying functional copies of the dystrophin gene (Duan et al., 2021). Early trials in animal models and initial human studies have shown promise, demonstrating the potential of stem cells to replace damaged skeletal muscles (Collins et al., 2005; Sacco et al., 2008; Cosgrove et al., 2014; Marg et al., 2014; Charville et al., 2015; Xu et al., 2015). However, stem cell therapies are not without their challenges and limitations. Specifically, graft rejection limits the effectiveness of cellular therapies as the host’s immune system targets transplanted stem cells and compromises their viability (Goldring et al., 2011; Goyenvalle et al., 2011; Maffioletti et al., 2014; Zhao et al., 2024).

One proposed solution to the issue of graft rejection has been the pretreatment of stem cells with conditions that improve their compatibility with the host. Various methods have been explored, including conditioning stem cells with oxidative stress using hydrogen peroxide (H₂O₂) or interleukin-4 (IL-4) to improve cell survival (Gargioli et al., 2018; Biressi et al., 2020). Another promising pretreatment method is hypoxia, or low oxygen conditions, which has been shown to enhance stem cell survival, proliferation, and therapeutic potential by modulating host immune responses and curtailing graft rejection (Luo et al., 2019; Ishiuchi et al., 2021; Pulido-Escribano et al., 2022; Wang et al., 2022). Moreover, hypoxia is known to upregulate Programmed Death Ligand 1 (PD-L1), a protein that plays a critical role in immune regulation (Ghosh et al., 2021; Chen et al., 2023; Handelsman et al., 2023). This relationship creates a unique opportunity to combine improved cell survival and immune-modulating effects of hypoxia conditioning with the immune evasive effects of PD-L1 upregulation. Thus, our novel strategy to mitigate graft rejection after cell transplantation involves hypoxia conditioning of muscle stem cells prior to implantation, with the aim of upregulating PD-L1 to suppress the host immune response.

To evaluate this strategy, we pre-conditioned muscle progenitor stem cells (MuSCs) with 5% hypoxia for 72 h and assessed hypoxia’s effect on the surface expression of major histocompatibility complex molecules, toll-like receptor (TLR) molecules, cell adhesion, and PD-L1. Additionally, we investigated preconditioning with interferon-γ (IFN-γ), an inflammatory cytokine that itself has shown potential in upregulating PD-L1 (Handelsman et al., 2023). Following conditioning with hypoxia and/or IFN-γ, intramuscular injections of MuSCs were performed on MDX (dystrophin-deficient) and C57BL/6J (wild-type) mice models to assess their immunogenicity and survival, both with and without a small-molecule inhibitor of PD-L1. Immunogenicity was measured by quantifying CD4^+^ and CD8^+^ T cell infiltration over time after cell transplantation in MDX mice, both with and without the PD-L1 inhibitor. MuSC survival was assessed using LacZ gene staining to compare hypoxia conditioning alone *versus* hypoxia conditioning combined with the same PD-L1 inhibitor. This comprehensive approach aims to improve the viability and effectiveness of stem cell therapies for muscular dystrophy by addressing the critical challenge of immune rejection.

## Methods and materials

### Animals

4-week-old C57BL/6J and C57BL/10ScSn-Dmdmdx/J (also known as MDX or dystrophin-deficient) male and female breeding pairs were purchased from Jackson Laboratory (Bar Harbor, ME) and bred to establish colonies for this study. All animal use was in accordance with National Institutes of Health guidelines and conformed to the principles specified in a protocol approved by Western Michigan University Homer Stryker M.D. School of Medicine Institutional Animal Care and Use Committee.

### Cell cultures and reagents

Mouse MuSCs (3-month-old, C57BL/6J female) were isolated according to Lavasani et al. (2013) and the *E. coli lacZ* gene was integrated into MuSCs (LacZ + MuSC) according to Naderian et al. (2011) using a lentivirus vector. LacZ + MuSC were maintained on collagen coated substrates with Dulbecco’s modified Eagle’s medium, high glucose (DMEM, Gibco), supplemented with 20% fetal bovine serum (FBS, Gibco), 1.5% chick embryo extract (APS, United Kingdom), and 1% penicillin/streptomycin at 37°C and 5% CO_2_. LacZ + MuSCs were subcultured with trypsin (0.25% trypsin-EDTA, Gibco BRL) at 50%–60% confluency and were used up to 30 passages.

### LacZ + MuSC characterization

Six biological replicates of LacZ + MuSCs were taken for RNA extraction after trypsinization. Total mRNA was isolated using the Monarch^®^ Total RNA Miniprep Kit (New England BioLabs, United States). The high- Capacity cDNA Reverse Transcription Kit (Applied Biosystems, United States) was used for the first strand cDNA synthesis. Quantitative PCR (qPCR) was performed with iTaq Universal SYBR Green Supermix (Bio Rad, United States) using the CFX Connect Real-Time System Thermal Cycler (Bio-Rad). All reactions were run in duplicate. After vortexing, 10 μL aliquots of the mixture were pipetted into each well of a 96-well thin-wall PCR plate (Bio-Rad). PCRs consisted of a denaturing cycle at 95°C for 3 min followed by 40 cycles of 10 s at 95°C, and 30 s at 60°C. Fluorescence was quantified during the 60°C annealing step and product formation was confirmed by melting curve analysis (65°C–95°C). Relative mRNA amounts of target genes were calculated after normalization to an endogenous reference gene, β-actin, and compared to the negative marker (CD45) with the arithmetic formula 1/ΔCt. Based on the cDNA sequences available at the EMBL database, the following specific murine primer pairs were designed by the software Primer3 (Whitehead Institute for Biomedical Research, Cambridge, MA, United States, http://www-genome.wi.mit.edu/cgibin/primer/primer3_www.cgi/) and confirmed by the sequences in the NCBI database (http://www.ncbi.nlm.nih.gov/). All primers shown in Supplementary Table S1 were synthesized by Integrated DNA Technologies (United States).

### Hypoxia conditioning

A hypoxia incubator (ThermScientific HERACellVIOS 160i, United States), set to 5% O_2_ nitrogen injection, was used to treat LacZ + MuSCs. Cells were treated for 6, 12, 24, 48, or 72 h under 5% O_2_ with 21% O_2_ as the control group. Additionally, LacZ + MuSCs were treated with hypoxia in conjunction with 0.03 ng/μL mouse IFN-γ Recombinant Protein (BioLegend, United States) or 1 μM BMS-1 (small-molecule inhibitor of PD-L1, MedChemExpress, United States).

### Western blotting

LacZ + MuSC cells were cultured in six-well plates and then lysed with 2x laemmli sample buffer solution (Bio Rad, United States) for the collection of total cellular protein. Each lane of a sodium dodecyl sulfate (SDS)-polyacrylamide gel was loaded with approximately 150 μg of extracted protein. SDS-Page was used to separate proteins by size which were then transferred to polyvinylidene fluoride (PVDF) membranes (Bio Rad, United States). The PVDF membranes were placed in a blocking buffer [TBS-T with 5% skimmed milk) then incubated at 25°C for 1 h. The same buffer was used to dilute the primary and secondary antibodies. Each PVDF membrane was incubated with primary and secondary antibodies sequentially. Finally, protein blots were developed using a SuperSignal West Pico PLUS Chemiluminesent Substrate ECL Plus kit (Thermo Scientific, United States)] and visualized using a chemiluminescence imager (LI_COR, United States). All Western blotting raw data in this paper are shown in Supplemental Materials. Antibodies used for Western blotting include: anti-PD-L1 (1:500, Abcam, ab213480, Boston, United States), anti-β actin (1:5,000, NB600-501). HRP-Goat Anti-Rabbit and HRP-Goat Anti-Mouse (Life Technologies, United States) were used as the secondary antibodies for anti-PD-L1 and anti-β actin, respectively.

### Intramuscular injection of MuSC in the murine gastrocnemius muscle

All mice received intramuscular injections to the gastrocnemius thigh muscles (GM). To prevent variation due to injection technique, a single, experienced technician performed intramuscular injections for all mice. Mice were anesthetized with isoflurane (5% induction, 1%–2% maintenance) delivered in oxygen by using an anesthetic vaporizer (Isoflurane, funnel-fill vaporizer, VetEquip, Piney River, VA). Once anesthetized, mice were placed sternally on a tabletop with a nose cone supplied with 1%–2% isoflurane gas to maintain anesthetic depth. Once a mouse was prepared for injection, the technician advanced the needle through the skin, within the marked area, and into the target muscle to a depth of approximately 2–4 mm. The syringe plunger was aspirated to check for inadvertent placement within a blood vessel, and then the injection was delivered. 2.0 × 10^6^ treated LacZ + MuSCs suspended in 20 µL of Hank’s Balanced Salt Solution (HBSS, Gibco) were injected into each GM.

### Gastrocnemius muscle isolation

Animals were sacrificed using carbon dioxide (CO_2_) inhalation followed by cervical dislocation as approved by the animal protocol. The GM was isolated according to Ionescu et al. (2016). Minced muscle tissue was digested using Collagenase Type II (ThermoFisher, United States) for 1.5 h at 37°C and 5% CO_2_ followed by Dispase II (ThermoFisher, United States) digestion for 1 h. After centrifugation at 3,500 rpm for 5 min, digested muscle tissue was mechanically dissociated in complete media (DMEM, 20% fetal bovine serum, 1.5% chick embryo extract, 1% penicillin/streptomycin) and filtered through a 70 µm filter. Isolated cells were plated in 6-well plates to be LacZ stained for LacZ + MuSCs survival after injection to the GM. Additionally, isolated cells were used for flow analysis to assess CD4^+^ and CD8^+^ infiltration after 3, 5, and 7d *in vivo* after GM injection.

### Flow cytometry analysis

An Attune NxT flow cytometer (Invitrogen, United States) was used for flow cytometric analysis. Antibodies included phycoerythrin (PE)-conjugated anti-mouse MHC-I (Catalog #562004, BD Biosciences, United States), TLR3 (Catalog #141904, BioLegend, United States) and TLR7 (Catalog #160004, BioLegend, United States); allophycocyanin (APC)-conjugated anti-mouse PD-L1 (Catalog #124312, BioLegend, United States), ICAM (Catalog #116120, BioLegend, United States) and CD4 (Catalog #100412, BioLegend); and Fluorescein isothiocyanate (FITC)-conjugated anti-mouse MHC-II (Catalog #562015, BD Biosciences, United States) and CD8 (Catalog #100706, BioLegend, United States). For cell surface analysis of MHC-I, MHC-II, TLR3, TLR7, ICAM, and PD-L1, both C2C12 and LacZ + MuSCs monolayers were grown for 72 h under 5% O_2_, 21% O_2_, 5% O_2_ with 0.03 ng/μL mouse IFN-γ, and 21% O_2_ with 0.03 ng/μL mouse IFN-γ before assessment with flow cytometry. CD4^+^ and CD8^+^ assessment was done on digested GM samples collected after 3, 5, 7d *in vivo* on injected LacZ + MuSCs treated for 72 h with 5% O_2_, 21% O_2_, 5% O_2_ with 1μM BMS-1, 21% O_2_ with 1 μM BMS-1 and 5% O_2_ with 0.03 ng/μL mouse IFN-γ, and 21% O_2_ with 0.03 ng/μL mouse IFN-γ. Spleen was collected as a positive control for CD4^+^ and CD8^+^ assessment. Medium fluorescent intensity results were assessed by Offline analysis with FlowJo 10.9.

### LacZ staining

LacZ gene expression was examined after fixing adherent cells (at 80% confluency) isolated from the GM muscle with 100% methanol for 5 min as described previously (Verma and Asakura, 2011).

### MTT assay

BMS-1 toxicity on the metabolic activity of LacZ + MuSC was analyzed using the MTT assay under normoxia conditions (21% O_2_). To assess cell viability for each BMS-1 concentration, 1 × 10^3^ lacZ + MuSC cells in 200 μL media were incubated with 1, 2, or 4 μM BMS-1 in a 96-well plate coated with collagen. After a 24 h incubation, 8 μL of a 5 mg/mL MTT 1-(4,5-Dimethylthiazol-2-yl)-3,5-diphenylformazan, Sigma #M2003) solution was added to each well. After 4 h, the cell-free supernatant was discarded, and the formazan crystals were dissolved with 100 μL DMSO. The OD was detected at 570 nM. The % viability rates were calculated relative to NAD(P)H-dependent cellular oxidoreductase activity in non-treated (CTL) *versus* BMS-1 treated cells.

### Statistical analysis

The data was presented as mean ± SD for flow cytometry analysis or mean ± SEM for western blot and MTT analysis. One-way ANOVA followed by Dunnett’s multiple comparisons test, and Unpaired or one-tailed t-test was performed (where applicable) using GraphPad Prism version 10.1.0. If not indicated otherwise, the criterion for significance was set at P ≤ 0.05. All **** represent P ≤ 0.0001 unless otherwise indicated.

## Results

### Hypoxia upregulates PD-L1 expression in skeletal muscle stem/progenitor cells

**FIGURE 1:**
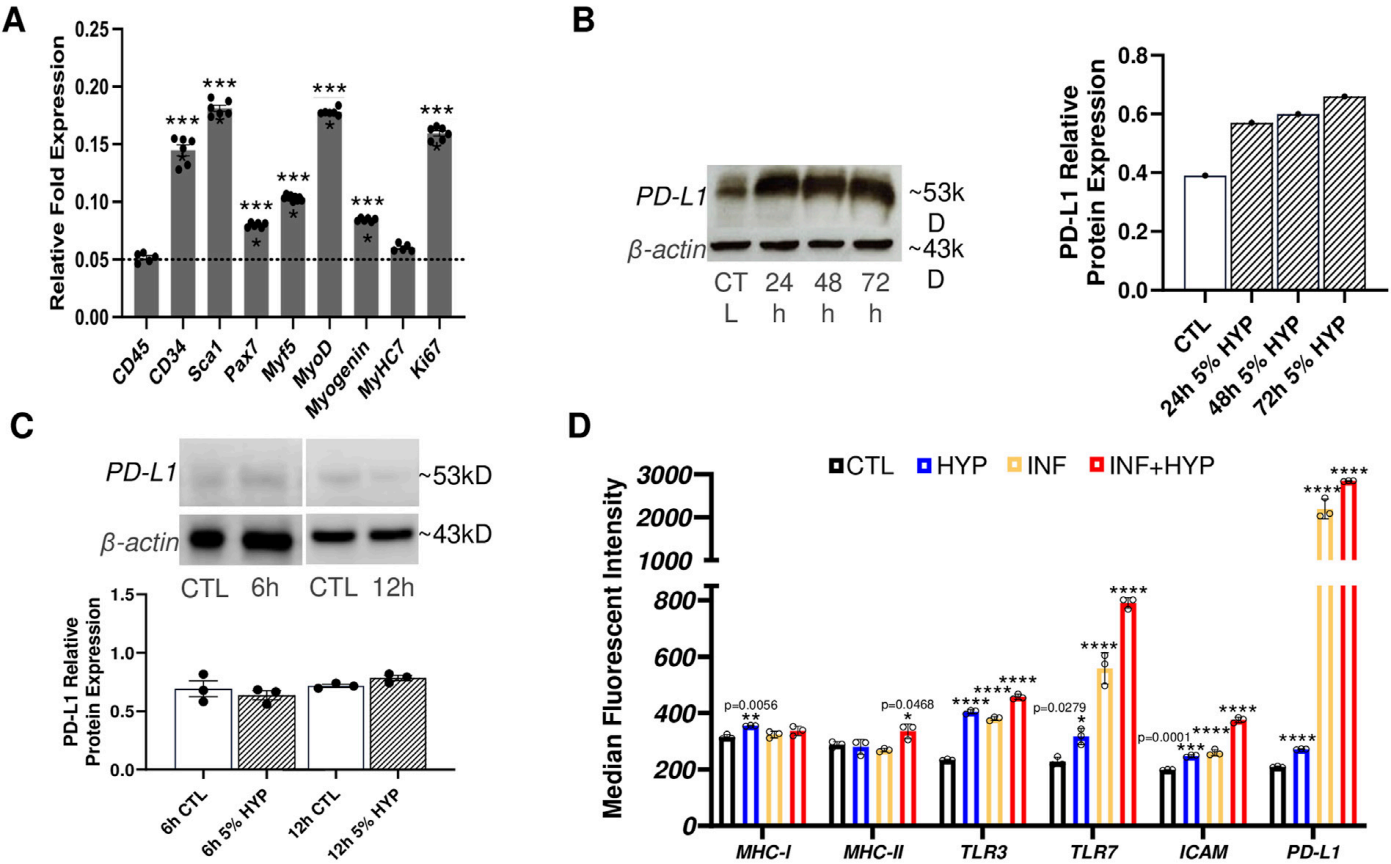
Characterization of PD-L1 expression and immunological surface markers with hypoxia conditioning in skeletal muscle stem/progenitor cells. **(A)** qPCR analysis of markers of skeletal muscle stem/progenitor cells (CD34, Sca1, Pax7, Myf5, MyoD, myogenin), marker of differentiated skeletal muscle (MyHC7), and cell proliferation (Ki67) used to characterize LacZ + MuSC. Data represented as n = 5–6 biological replicates with means ± SEM and was analyzed with Ordinary one-way ANOVA followed by Tukey’s multiple comparison test. **(B)** Quantitative and semi-quantitative results of PD-L1 protein expression after 24h, 48h, and 72 h hypoxia conditioning compared to normoxia treated cells (CTL). Data represented as n = 1. **(C)** Quantitative and semi-quantitative analysis of PD-L1 relative protein expression in LacZ + MuSC after 6h and 12 h hypoxia conditioning. Data represented as n = 3 biological replicates with means ± SEM and was analyzed with Unpaired t-test. **(D)** LacZ + MuSCs treated for 72 h with 5% HYP, HYP with 0.03 ng/μL IFN-γ, 21% O_2_ (CTL), or 21% O_2_ with 0.03 ng/μL IFN-γ. Surface expression levels of MHC-I, MHC-II, TLR3, TLR7, ICAM, and PD-L1 were assessed with Flow Cytometry in each treatment population. Data represented as n = 3 biological replicates with means ± SD and was analyzed with Ordinary one-way ANOVA followed by Tukey’s multiple comparison test.

Myoblast transfer therapy was the first form of quasi-gene therapy proposed for treating DMD (Partridge, 2002). However, skeletal muscle stem/progenitor cells are more valuable given their enhanced regenerative capacity and their improved engraftment potential (Hosoyama et al., 2012). Thus, we first isolated MuSC and integrated the *E. coli lacZ* gene to these cells (LacZ + MuSCs). We then used qPCR to quantify the levels of the following markers: CD34, a hematopoietic/progenitor marker; Sca1, a muscle derived stem cell marker (MDSC); Pax7, a MuSC maintenance marker; Myogenic factor 5 (Myf5), a satellite cell marker; myoblast determination protein 1 (MyoD), an active satellite cell marker; Myogenin, an early muscle skeletal muscle differentiation marker; Myosin Heavy Chain 7 (MyHC7), a skeletal muscle marker; and Ki67, a proliferation marker (Figure 1A). These markers were compared to the negative control, CD45, a marker of nucleated hematopoietic cells. The qPCR results showed significant (****P ≤ 0.0001) muscle stem/progenitor related transcriptome levels of CD34, Sca1, Pax7, Myf5, MyoD, and myogenin, compared to the negative marker, CD45, whose baseline was indicated with a dotted line. Low levels of CD45 expression confirm the population of MuSCs used for analysis was indeed composed purely of muscle-derived cells and not contaminated with immune cells of hematopoietic origin (Testa et al., 2020). Additionally, no significance in the MyHC7 marker indicated that our LacZ + MuSCs were not undergoing further differentiation during proliferation, as indicated by their significantly high transcriptome level of Ki67 (****P ≤ 0.0001). Next, we assessed the LacZ + MuSCs ability to upregulate PD-L1 using increasing exposure time to 5% hypoxia (HYP) compared to normoxia (CTL). Western blot analysis indicated increasing PD-L1 protein expression up to 72 h (Figure 1B). To determine if LacZ + MuSCs were able to upregulate PD-L1 at an earlier time point during hypoxia conditioning, we compared PD-L1 protein expression levels after 6h and 12 h (Figure 1C). Semi-quantitative analysis of PD-L1 relative protein expression normalized to the β-actin control indicated that MuSC started upregulating PD-L1 expression as early as 12 h and peaked close to 72 h. Given these findings, all further hypoxia conditioning was done for a minimum of 72 h to maximize PD-L1 expression for further investigation. Finally, to determine the immunological capacities of hypoxia conditioned LacZ + MuSCs *in vitro*, we investigated the cell surface expression with flow cytometry of MHCI/II, Toll-like receptors 3 and 7 (TLR3, TLR7), ICAM, and PD-L1 after a 72 h treatment with normoxia (CTL), 5% hypoxia (HYP), IFN-γ (INF) alone, and INF with 5% hypoxia (INF + HYP) (Figures 1D, E). We showed that cell surface expression of MHC-I, MHC-II, TLR3, and TLR7 in MuSC was similar under normoxia and hypoxia conditions (Figure 1D). However, MHC-II was significantly upregulated with IFN-γ with and without hypoxia (Figure 1D). Furthermore, a calculated 7.1 and 9.5-averaged fold change of PD-L1 cell surface expression was seen with IFN-γ and IFN-γ plus hypoxia treated LacZ + MuSCs, respectively, in comparison to hypoxia treatment alone.

### Hypoxia induced PD-L1 protein expression was maintained for 12 h in normoxia

**FIGURE 2:**
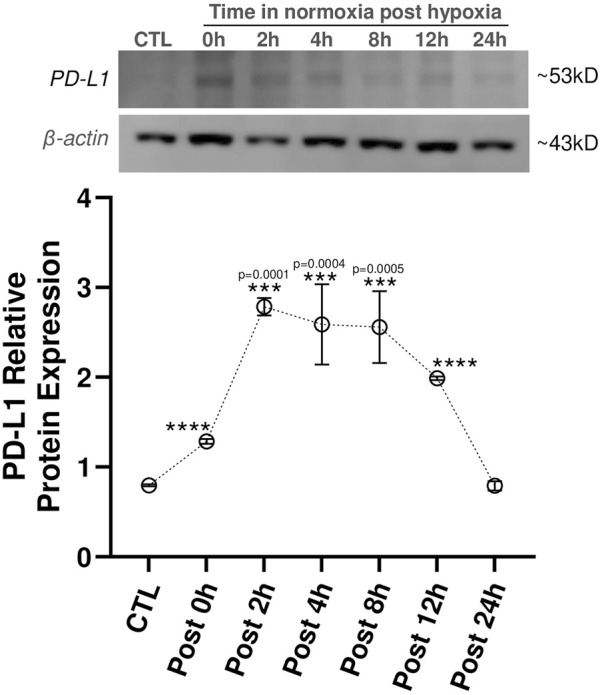
PD-L1 expression maintained in skeletal muscle progenitor/stem cells until 12 h post hypoxia conditioning. MuSC + LacZ were treated for 72 h with 5% hypoxia (HYP) and then transferred to normoxia (CTL, 21% O2) for Post 0 h, 2 h, 4 h, 8 h, 12 h, or 24 h. Data represented as n = 3 biological replicates for each cell type with means ± SEM and was analyzed with Ordinary one-way ANOVA followed by Tukey’s multiple comparison test.

Given that we saw PD-L1 upregulation in LacZ + MuSCs after 72 h with 5% hypoxia conditioning, we sought to discover how long PD-L1 would stay upregulated after the conditioned cells were placed back into normoxia conditions (Figure 2). First, we confirmed that PD-L1 relative protein expression was upregulated (****P ≤ 0.0001) after the 72 h hypoxia conditioning (Post 0 h) in comparison to the baseline cells conditioned in normoxia for 72 h (CTL). Furthermore, a calculated 0.62 average fold increase in PD-L1 protein expression was seen with 72 h hypoxia conditioning compared to 72 h normoxia conditioned LacZ + MuSCs. Interestingly, PD-L1 expression increased and plateaued from 2 to 8 h under normoxia conditions (Post 2h, Post 4h, and Post 8 h) before decreasing to baseline levels by 24 h. Average fold change in PD-L1 expression was 2.5, 2.25, and 2.22 for Post 2h, Post 4h, and Post 8 h in comparison to the 72 h normoxia conditioned LacZ + MuSCs, respectively. This data indicated that LacZ + MuSCs were able to maintain hypoxia induced PD-L1 expression even under normoxia, mitigating the need for a continuous hypoxic environment to harvest the cells for cell transplantation procedures.

### PD-L1 upregulation with hypoxia decreased T-Cell CD4^+^ and CD8^+^ cell infiltration

**FIGURE 3:**
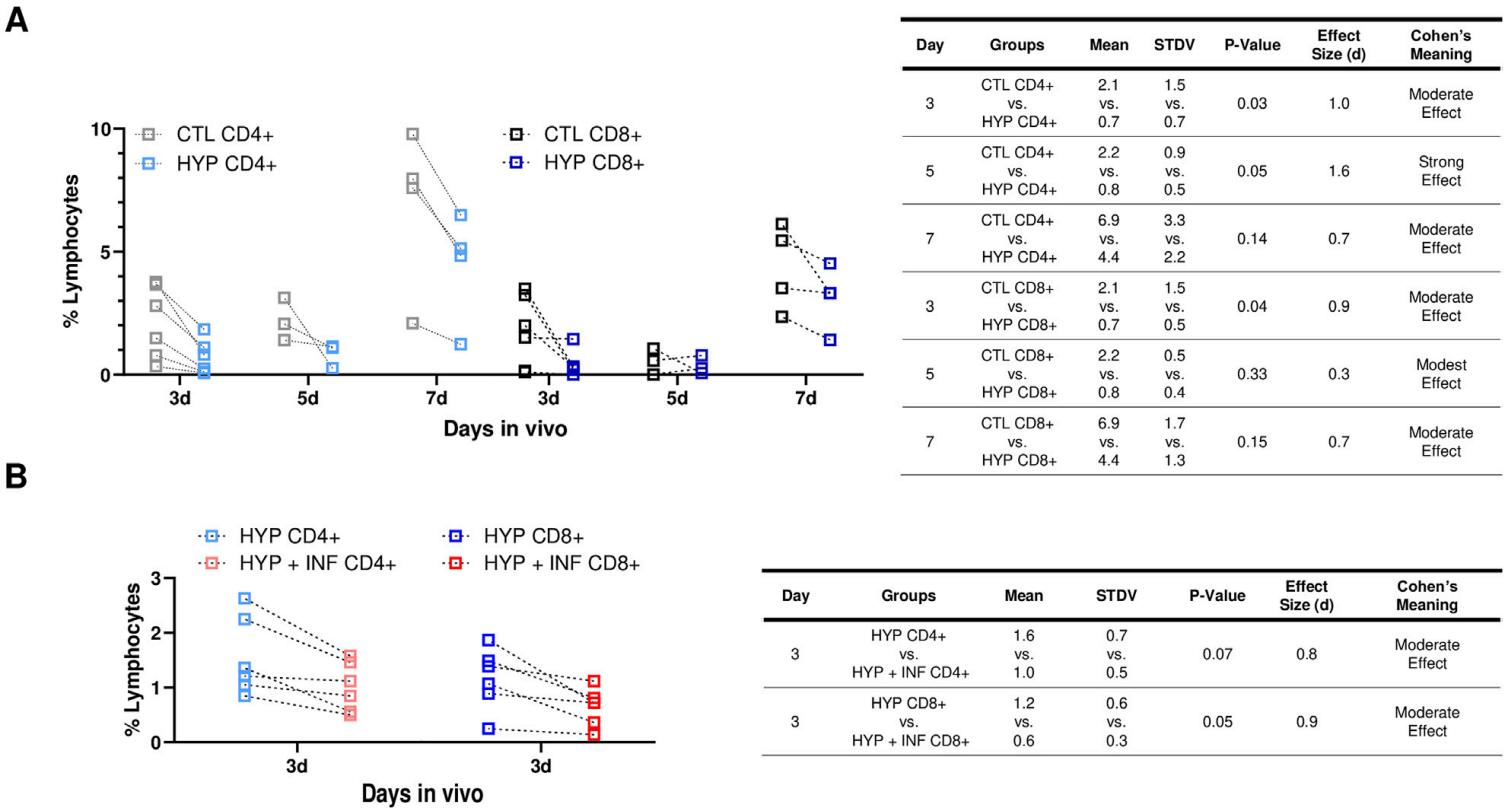
Hypoxia induced PD-L1 upregulation in LacZ + MuSCs decreased lymphocyte infiltration and improved cell survival after cell implantation. **(A)** Decreases seen in CD4^+^ and CD8^+^ cells present in GMs on days 3, 5, and 7 after injection with 72 h hypoxia conditioned LacZ + MuSCs (HYP CD4^+^ or CD8^+^) compared to matched GM injected with 72 h normoxia conditioned LacZ + MuSCs (CTL CD4^+^ or CD8^+^). Mean, standard deviation, *p*-value, effect size (d), and Cohen’s meaning were shown in the table for each hypoxia group compared to the normoxia group each day *in vivo*. Dotted connected lines per sample represented GM matched samples per injected mouse. Data represents n = 3-6 biological replicates for each day *in vivo* and was analyzed with one-tailed t-test. **(B)** Decreases in CD4^+^ and CD8^+^ cells present in GMs 3d after injection with 72 h hypoxia and 0.03 ng/μL IFN-γ conditioned LacZ + MuSCs (HYP + INF CD4^+^ or CD8^+^) compared to matched GM injected with 72 h hypoxia conditioned LacZ + MuSCs (HYP CD4^+^ or CD8^+^). Mean, standard deviation, *p*-value, effect size (d), and Cohen’s meaning were shown in the table for each hypoxia + INF group compared to the HYP group *in vivo*. Dotted connected lines per sample represented GM matched samples per injected mouse. Data represents n = 6 biological replicates *in vivo* and was analyzed with one-tailed t-test.

Infiltration of CD4^+^ and CD8^+^ T cells play a major role in acute allograft rejection, as well as increase memory cells associated with long-term allograft dysfunction (Yap et al., 2015). Thus, we evaluated the percent CD4^+^ and CD8^+^ cell infiltration in C57BL/J6 (6–9 months, male and female, n = 13) with both 72 h hypoxia conditioned and normoxia conditioned LacZ + MuSCs implanted separately into the left and right GMs of each mouse. These treated muscles were harvested for cell isolation 3, 5, and 7 days and stained for CD4^+^ and CD8^+^ cells for Flow Cytometry (Figures 3A, B). A rigorous gating strategy of SSC-W vs. SSC-H, FSCH vs. FSC-A, and SSC-A vs. FSC-A was used to isolate singlets in the population (Supplementary Figure S4). This same gating strategy was applied to CD4^+^ and CD8^+^ cells isolated from hypoxia conditioned and hypoxia conditioning with IFN-γ injected muscle samples (Supplementary Figure S4). A spleen sample was used to confirm CD4^+^ and CD8^+^ positive cell populations while unstained samples were used as negative controls. Our results suggest that CD4^+^ and CD8^+^ cell infiltration was decreased across 3d, 5d, and 7d with hypoxia (HYP CD4^+^ or CD8^+^) conditioned injected muscles *versus* matched, normoxia (CTL CD4^+^ or CD8^+^) conditioned injected muscles (Figure 3B). The table shows the mean, standard deviation (STDV), and *p*-value from two tailed student t-tests for each group. Additionally, Cohen’s d effect size was calculated as the difference between the mean of two groups and then divided by the standard deviation of the control group (Lakens, 2013). The commonly used interpretation was to refer to effect sizes as weak effect (d = 0–0.20), modest effect (d = 0.21–0.50), moderate effect (d = 0.50–1.00), and strong effect (d = >1.00) (Cohen, 2013). HYP CD8^+^ injected muscles showed a similar decrease in CD8^+^ T-cells across 3d, 5d, and 7d compared to matched CTL CD8^+^ injected muscles. The table showed the mean, standard deviation (STDV), and *p*-value from two tailed student t-tests for each group. Additionally, Cohen’s d effect size was calculated as the difference between the mean of two groups and then divided by the standard deviation of the control group. Overall, we saw a statistically significant (*p* < 0.05) decrease and a moderate Cohen’s effect in CD4^+^ and CD8^+^ cells 3d after injection compared to 5d and 7d post injection. Thus, any further experiments involving CD4^+^ and CD8^+^ infiltration were limited to 3d post injection.

We then explored whether 72 h hypoxia and IFN-γ conditioned LacZ + MuSCs implanted separately to the GMs would have any further effects in CD4^+^ and CD8^+^ infiltration after 3d compared to the matched GM implanted with 72 h hypoxia conditioned LacZ + MuSCs. This was based on our previous observation that showed significantly high surface expression levels of PD-L1 in LacZ + MuSCs when conditioned with hypoxia and 0.03 ng/μL IFN-γ for 72 h. C57BL/J6 mice, 3 months, male and female (n = 6) were used for this experiment.

We observed a similar range of percent lymphocytes of HYP CD4^+^ and CD8^+^ cells (Figure 3D) compared to the batch of 3–9-month-old mice (Figure 3B) indicating that the age of the mice did not impact the percent CD4^+^ and CD8^+^ lymphocytes present in the GMs. Interestingly, we also saw a further decrease in CD4^+^ and CD8^+^ lymphocyte infiltration with the hypoxia and IFN-γ injected GMs (HYP + INF-γ CD4^+^ and CD8^+^) in comparison to the matched hypoxia conditioned GMs (Figure 3D). More specifically, CD8^+^ cells were significantly decreased (*p* = 0.05) with injected hypoxia and IFN-γ conditioned LacZ + MuSCs compared to CD4^+^ cells. Cohen’s effect size (d) indicated that injected hypoxia and IFN-γ conditioned LacZ + MuSCs had a moderate effect in reducing CD4^+^ and CD8^+^ infiltration. These results added further evidence that the higher levels of PD-L1 upregulation seen after hypoxia and IFN-γ conditioning have increased effects in reducing T-cell infiltration after cell transplantation.

### Hypoxia induced upregulation of PD-L1 is a major factor involved in decreased CD4^+^ and CD8^+^ T-Cell infiltration and increased graft survival after cell implantation

**FIGURE 4:**
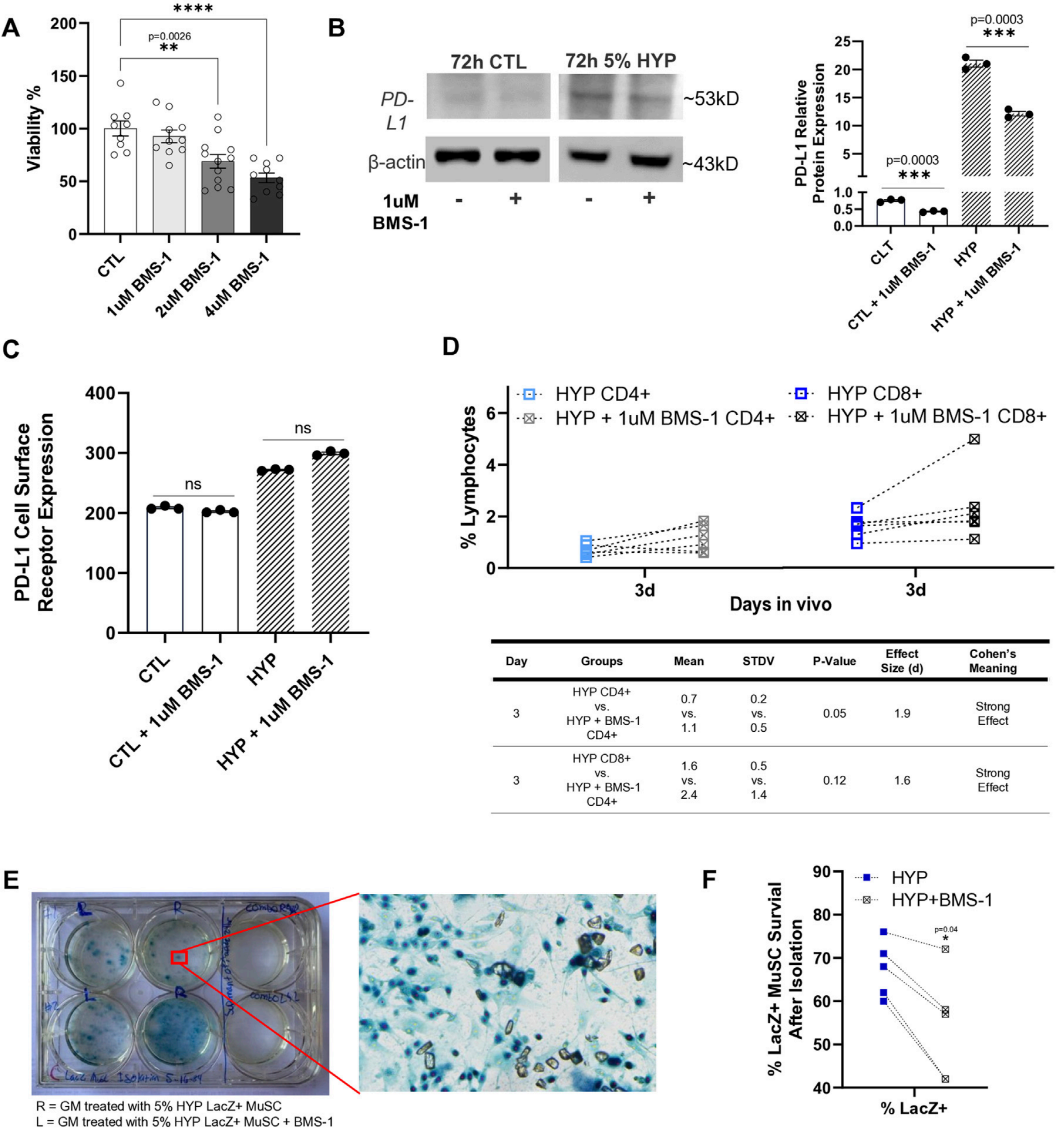
Small molecule inhibitor, BMS-1, further supports role of hypoxia-induced upregulation of PD-L1 in reducing T-cell infiltration after cell implantation. **(A)** Viability percent of LacZ + MuSC after 24 h treatment with increasing concentrations of BMS-1 in normoxia conditions. Data represented as n = 9–12 biological replicates with means ± SEM and was analyzed with Ordinary one-way ANOVA followed by Tukey’s multiple comparison test. **(B)** PD-L1 relative protein expression levels normalized to β-actin showed significant decreases in PD-L1 expression with 72h, 1 μM BMS-1 treatment as a result of competition with the anti-PD-L1 IgG antibody. Data represented as n = 3 biological replicates with means ± SEM and was analyzed with Unpaired t-test. **(C)** Flow Cytometry analysis of surface level PD-L1 expression in LacZ + MuSC indicating similar cell surface receptor expression of PD-L1 with and without 72 h BMS-1 treatment after normoxia and hypoxia conditioning. Data represented as n = 3 biological replicates with means ± SEM and was analyzed with Ordinary one-way ANOVA followed by Tukey’s multiple comparison test. **(D)** Increases in CD4^+^ and CD8^+^ cells present in GMs 3d after injection with 72 h hypoxia conditioned LacZ + MuSCs injected with 1uM BMS-1 (HYP + 1uM BMS-1 CD4^+^ or CD8^+^) compared to matched GM injected with 72 h hypoxia conditioned LacZ + MuSCs (HYP CD4^+^ or CD8^+^). Mean, standard deviation, *p*-value, effect size (d), and Cohen’s meaning were shown in the table for each hypoxia group injected with or without BMS-1 *in vivo*. Dotted connected lines per sample represented GM matched samples per injected mouse. Data represents n = 6 biological replicates *in vivo* and was analyzed with one-tailed t-test. **(E)** Representative image of surviving LacZ + cells from treated GM shown as having dark blue nuclei. **(F)** Percent survival of LacZ + MuSCs counted in matched GM muscles injected with 5% condition LacZ + MuSC or 5% condition LacZ + MuSC mixed with 1 μM BMS-1 prior to injection. Data represented as n = 5 biological replicates and was analyzed with one-tailed t-test.

BMS-1 is considered a small molecule inhibitor of PD-L1 due to its ability to bind to PD-L1, thereby blocking its interaction with PD-1. This binding is thought to alleviate the inhibitory effect of soluble PD-L1 on T-cell receptor-mediated activation of T-lymphocytes (Skalniak et al., 2017). Since there were no references on BMS-1’s effect or toxicity on skeletal muscle stem/progenitor cells, we sought to confirm an optimal concentration to use to treat our LacZ + MuSCs. First, we tested the toxicity of increasing concentrations of BMS-1 after 24 h treatment on the LacZ + MuSCs using the MTT assay under normoxia conditions (Figure 4A). The results showed that BMS-1 concentrations above 1 μM had dose dependent negative effects on the percent viability of our LacZ + MuSCs. Thus, 1 μM BMS-1 was considered a safe, maximum dose and was used for all experiments from this point forward. A previous publication recommended a 72 h treatment on natural killer cells and HepG2 cells with BMS-1 to disturb the interaction between PD-1 and PD-L1 (Zhao et al., 2019). Given that the 72 h BMS-1 treatment aligned with our 72 h hypoxia conditioning, we decided to utilize 72 h as our minimum treatment time for both *in vivo* and *in vitro* experiments. Thus, we investigated the ability of BMS-1 to compete with the anti-PD-L1 IgG antibody through western blot analysis (Figure 4B). The results showed that BMS-1’s inhibitory capability was almost half in both normoxia and hypoxia conditioned LacZ + MuSCs after 72 h. The average fold change of PD-L1 protein expression in the presence of BMS-1 was 43% and 42% less after 72 h normoxia and hypoxia conditioning, respectively. To prove that BMS-1 does not affect PD-L1 transcriptome expression, we measured the cell surface PD-L1 receptor expression (medium fluorescent intensity) after 72 h treatment with BMS-1 in normoxia and hypoxia conditions using Flow Cytometry (Figure 4C). We noted that surface expression of PD-L1 in LacZ + MuSCs after hypoxia conditioning was similar to our previous results in Figure 1D. Additionally, the results showed no statistical differences in PD-L1 surface expression between cells treated and not treated with BMS-1 under normoxia and hypoxia conditions, implying BMS-1’s antagonistic effect on the PD-L1 receptors themselves and not the PD-L1 expression at the transcriptional and translational levels.

Finally, to determine if PD-L1 upregulation was a factor of reduced CD4^+^ and CD8^+^ T-cell infiltration after cell implantation to the GMs, LacZ + MuSCs were conditioned for 72 h with 5% hypoxia. Prior to injection to one side of the GM per mouse, 1 μM BMS-1 was added to 2 × 10^6^ of the hypoxia treated cells while the other GM of the same mouse received 2 × 10^6^ hypoxia treated cells to act as a control. MDX (3 months, male and female, n = 6) were used for this experiment. After 3d *in vivo* the GMs of matched mice were isolated to evaluate CD4^+^ and CD8^+^ infiltration with and without BMS-1 on LacZ + MuSCs conditioned with hypoxia. The same rigorous gating strategy of SSC-W vs. SSC-H, FSCH vs. FSC-A, and SSC-A vs. FSC-A was used to isolate CD4^+^ and CD8^+^ singlets from each GM sample as mentioned before (Supplementary Figure S6). A spleen sample was used to confirm CD4 and CD8 positive cell populations while unstained samples were used as negative controls. We showed that the addition of 1 μM BMS-1 increased the presence of CD4^+^ and CD8^+^ cells in the GM 3d post injection (Figure 4D). Furthermore, CD4^+^ cells were significantly increased in the GM (*p* = 0.05) with the addition of BMS-1 compared to GMs injected with hypoxia conditioned LacZ + MuSCs alone. Finally, Cohen’s effect size (d) indicated a strong effect in the percent population of CD4^+^ and CD8^+^ cells in GMs injected with the BMS-1 small molecule inhibitor.

To estimate the ability of skeletal muscle stem/progenitor cell survival after cell transplantation, LacZ + MuSCs were conditioned with 5% hypoxia for 72 h. The same number (2 × 10^6^) of both LacZ + MuSCs were implanted separately into the GM, except that one set of cells was mixed with 1 μM of BMS-1 prior to the injection. MDX (2–3 months, male and female, n = 5) mice were used for this experiment. These muscles were then harvested for cell isolation 4 days after injection and subsequently evaluated for LacZ + cell survival by counting dark blue nuclei (Figure 4D). Counting was performed by capturing five microscopic images taken from random portions of each cell culture plate well. Our results showed that the hypoxia-conditioned MuSCs had an improved survival rate compared to the MuSCs injected with BMS-1 as indicated by a *p*-value = 0.04 using one-tailed t-test (Figure 4E).

## Discussion

Since the early 1990s, progenitor cell transplantation has been proposed as a treatment for Duchenne muscular dystrophy (DMD) (Mouly et al., 2005). Initial proof-of-concept studies involving intramuscular injections of myoblasts into MDX mice showed promising results, with the formation of hybrid, dystrophin-expressing muscle fibers (Skuk and Tremblay, 2003). Encouraged by these outcomes, human clinical trials were conducted, wherein DMD patients received intramuscular injections of allogeneic myoblasts from partially immune-compatible donors (Arjona et al., 2022). However, these trials largely failed to demonstrate clinical benefits. In fact, the majority of patients showed minimal functional improvement in the recipient limbs, as evidenced by muscle biopsies revealing scant dystrophin expression. This lack of clinical improvement is likely due to host immune rejection, which impairs myoblast migration and survival (Bentzinger et al., 2014). Various strategies have been explored to address this issue, yet none have achieved significant clinical relevance due to decreased *in vivo* migration and proliferation of muscle stem cells. Thus, the persistent challenge of host immune rejection remains a major obstacle, highlighting the need for innovative approaches to enhance the viability and effectiveness of stem cell therapies for muscular dystrophy.

Progenitor muscle stem cells are superior to myoblast cells for transplantation due to their enhanced regenerative capacity, improved engraftment, and greater plasticity, allowing better adaptation to the host tissue (Usas and Huard, 2007). They also have reduced immune rejection, are more amenable to gene correction, and support long-term muscle repair and maintenance (Feige et al., 2018). Additionally, muscle-derived stem cells have been shown to preserve their phenotype up to 200 population doublings and exhibit similar skeletal muscle regeneration at low and high population doublings after cell transplantation (Deasy et al., 2005). These advantages make progenitor muscle stem cells a more promising option for treating muscular dystrophies and other muscle-related disorders. Regarding these advantages, we chose to isolate and characterize mouse MuSC from C57BL/6J mice for the utilization of this research. Our resulting characterization with qPCR confirmed our cultured MuSCs had a stable muscle stem/progenitor lineage which was tested over 6 different passage points.

Moreover, the improved short-term survival of these MuSCs demonstrated in our experiments may offer benefits beyond their inherent replicative potential. MuSCs are inherently immunomodulatory and exhibit anti-inflammatory effects (Rigamonti et al., 2014; Klimczak et al., 2018). Upon transplantation into the host, they activate anti-inflammatory pathways and express angiogenic factors like vascular endothelial growth factor (VEGF). This activation leads to the migration of host stem cells to the injury site (i.e., where the exogenous stem cells are injected), which secrete monocyte chemoattractant protein-3, SDF-1, and IL-8, creating a more hospitable environment for the exogenous stem cells to engraft and replicate (Wang et al., 2007). Thus, exogenous stem cells stimulate host stem cell homing to their location. This interaction is further enhanced by the exogenous stem cells’ secretion of insulin-like growth factor (IGF-1) and hepatocyte growth factor (HGF) (Mangi et al., 2003). Consequently, the interactions between transplanted stem cells and the host are multifaceted, with some being supportive to the transplanted cells. This beneficial interplay contributes to the prolonged survival of the transplanted stem cells.

Under hypoxic conditions, Hypoxia-Inducible Factor 1-α (HIF-1α) is stabilized and activates various genes, including PD-L1 (Shurin and Umansky, 2022). Studies have shown that hypoxia-induced upregulation of PD-L1 can occur relatively quickly, often within 24–48 h under 1%–3% oxygen levels (Liu et al., 2012; Bailey et al., 2022; van Duijn et al., 2022). We chose to utilize 5% hypoxia for conditioning our MuSC given that 5% hypoxia has been shown to enhance survival and proliferation, as well as promote adaptive responses by reducing oxidative stress compared to severe hypoxia (e.g., <1% oxygen) (Liu et al., 2012; Redshaw and Loughna, 2012; Elashry et al., 2022). Additionally, mild hypoxia (around 5% oxygen) promotes the secretion of pro-angiogenic factors, such as VEGF, leading to improved integration of transplanted cells and tissue repair (Deasy et al., 2009; He et al., 2021; Nguyen et al., 2021).

Given the relationship between hypoxia’s enhancement properties on MuSC, and hypoxia’s influence on increasing PD-L1 transcription, we utilized western blot analysis to determine an optimal duration of 5% hypoxia conditioning that resulted in the highest PD-L1 protein expression in our MuSCs. Our results indicated that PD-L1 expression increases up to 72 h in our MuSCs. We did not test beyond 72 h due to increased cell proliferation, leading to nutrient limitation and increasing acidic conditions. PD-L1 expression under hypoxia was also assessed at short time frames, 6h–12 h. However, our results indicated that shortened hypoxia exposure failed to significantly upregulated PD-L1. Thus, a 72 h hypoxia conditioning was optimal for high PD-L1 expression in the MuSC and was utilized for further experimentation. Literature has shown that prolonged hypoxia treatments, such as 72h, have more significant benefits on muscle stem cells, satellite cells, and muscle progenitor cells compared to short-term (≤24 h) treatment. For example, 24 h hypoxia treatments can initially elevate hypoxia-inducible factors (HIF-1α and HIF-2α) while prolonged hypoxia causes a hypoxia-inducible factor expression switch from HIF-1α to the prominently stable HIF-2α, which plays a significant role in long-term cell adaptation (Blum et al., 2016). Evidence of improved muscle regeneration has been seen with muscle progenitor cells and C2C12 myoblast treated with prolonged hypoxia exposure as a result of improved myogenic differentiation and fusion into mature muscle fibers through better cellular adaptation (Elashry et al., 2022; Pircher et al., 2022). Also, the effects of 24 h hypoxia on C2C12 myoblast was shown to not be effective in improving the proliferation rate, viability, nor transcription factor expression during myogenesis (Sharkey, 2020). Thus, a 72 h hypoxia conditioning was optimal for high PD-L1 expression in our MuSC and was utilized for further experimentation given its proven enhancement in stabilized, cellular adaptation.

Exposure to hypoxia can influence the expression of various immune-related, cell surface markers in muscle stem cells, affecting their function and interactions with immune cells within the tissue (Wobma et al., 2018; Di Mattia et al., 2021). Thus, in addition to PD-L1, we explored the effects that hypoxia conditioning might have on surface expression of immune related markers, MHC I, MHC II, TLR3, TLR7, and ICAM, in the MuSCs. MHC I expression in particular has been shown to be abnormal in patients with different types of myopathies and neurogenic disorders (Milisenda et al., 2023). MHC I is also strongly expressed in myoblast cells, while its higher expression in embryonic stem cells compared to muscle-derived stem cells suggests better transplantation capacity, as muscle-derived stem cells are less likely to trigger lymphocyte infiltration. (Roy et al., 1991; Qu-Petersen et al., 2002). With hypoxia conditioning alone, there was a statistically significant increase in MHC I cell surface expression. Our results showed a decrease in the percent CD4^+^ and CD8^+^ T cell infiltration after intramuscular injection suggesting that this upregulation of MHC I on our MuSCs after hypoxia conditioning, in conjunction with PD-L1 upregulation, prevented tissue infiltration by CD4^+^ and CD8+T cells.

MHC II molecules are known to present antigens derived from extracellular proteins to CD4^+^ T lymphocytes (Roche and Furuta, 2015). In our experiments hypoxia conditioning did not affect MHC II expression in MuSCs, while still dampening a CD4^+^ T cells infiltrative response. This provide some evidence that there is an alternative mechanism in addition to PD-L1 which is prompting this diminished immune infiltration, as traditionally MHC II is required to enact the co-inhibitory effects prompted by PD-L1 (Handelsman et al., 2023). It was also noted that our results showed a significant increase in MHC II surface expression in MuSCs conditioned with hypoxia plus IFN-γ. This was accompanied by a further decrease in the percent CD4^+^ and CD8^+^ T cell infiltration after intramuscular injection with MuSCs conditioned with hypoxia plus IFN-γ compared to hypoxia conditioning alone. These results suggest that IFN-γ has an additive immune-privilege effect because of its ability to significantly increase cell surface expression of PD-L1 as well as MHC II in our MuSCs compared to hypoxia alone. A common dosage range of 0.01–0.1 ng/μL of IFN-gamma has been used to induce PD-L1 expression in cancer models and immune cells such as macrophages (Chen et al., 2019; Knopf et al., 2023). However, there is limited direct evidence on specific concentrations of IFN-gamma used in *in vitro* studies on muscle stem cells, satellite cells, or muscle progenitor stem cells. Studies involving muscle regeneration often use IFN-gamma concentrations in the range of 0.01–0.1 ng/μL to induce various immune responses which can affect muscle repair and satellite cell function during regeneration. For example, in the context of the immune system, inflammation, and muscle regeneration, IFN-gamma has been shown to promote myoblast proliferation, but inhibit myogenic differentiation *in vitro* given a reduction in the myosin heavy chain content (Grzelkowska-Kowalczyk et al., 2015; Yang and Hu, 2018). However, specific references directly relating IFN-gamma to muscle stem cells in the context of PD-L1 expression were lacking in the literature. In our study we proceeded with using 0.03 ng/μL of IFN-gamma which was within the range of 0.01–0.1 ng/μL seen in the literature and our LacZ + MuSC treated with IFN-gamma alone or IFN-gamma plus 5% hypoxia showed a 7.1 and 9.5 average fold increase in PD-L1 cell surface expression, respectively. These average fold expression results were well above the 1.5 fold-change, *p* < 0.05 requirements declared by Peart et al. (2005) and Raouf et al. (2008) to meet differential expression compared to the normoxia, control group (Peart et al., 2005; Raouf et al., 2008).

TLRs regulate the crosstalk between the innate and adaptive immune systems and are expressed on various stem/progenitor cells (Sallustio et al., 2019). In these cells, TLRs influence motility, proliferation, differentiation, self-renewal, and immunomodulation, playing a critical role in the response to tissue damage and reparative processes (Mastri et al., 2012; Rashedi et al., 2017; Mekhemar et al., 2020; Tingstad et al., 2021). It is recognized that sustained TLR pathway activation in muscular dystrophy enhances further tissue damage leading to fiber loss and increased inflammation (De Paepe, 2020). It is also understood that inflammatory myopathies express TLR3 near areas of infiltrating mononuclear cells (Schreiner et al., 2006). Additionally, TLR7 ligand ssRNA has been shown to induce inflammation in mdx mice (Henriques-Pons et al., 2014). Our results showed significant cell surface expression of TLR3 and TLR7 on our MuSCs with hypoxia conditioning. However, the focus of our current study was primarily related to an adaptive immune response rather than an innate one so evaluating the role TLR3 and TLR7 was beyond the scope of this paper but warrants further investigation.

ICAM is a cell surface protein expressed on many cell types, including muscle stem cells and satellite cells, and is responsible for muscle regeneration (Dearth et al., 2013; Goh et al., 2015; Pizza et al., 2017). However, this protein is also known to interact and retain leukocytes, particularly in endothelial cells by increasing trans-endothelial migration of leukocytes. We noted that surface ICAM was significantly expressed compared to basal levels on our MuSC conditioned with hypoxia as well as on MuSCs conditioned with hypoxia and IFN-γ. Given that we did not see increased CD4^+^ and CD8^+^ T cell infiltration after intramuscular injection of hypoxia conditioned MuSCs, our results suggest that higher expression levels of ICAM on our MuSCs did not facilitate adhesion, migration, nor activation of T cells through the lymphocyte function-associated antigen 1 and ICAM (Marlin and Springer, 1987). However, similar to TLR3 and TLR7, hypoxia and IFN-γ′s effects on the innate immune response may be better characterized in future studies.

Host-mediated immune rejection against transplanted allogeneic MuSCs is complex, involving both innate and adaptive immune mechanisms. However, a primarily driver in immune rejection that has been identified is T-cell mediated induced histo-incompatibility (Tremblay et al., 2009; De Paepe and De Bleecker, 2013; Cruz-Guzmán et al., 2015). Systemic immunosuppressants, such as Tacrolimus, are commonly used to mitigate this response, but their long-term use is limited by potential infectious complications (Skuk et al., 2004; Tremblay et al., 2009). Given these immunologic limitations on transplanted MuSCs, our experiments demonstrate how hypoxia conditioning of grafted cells may prevent these previously unchecked T cell-driven responses. Hypoxia-conditioned MuSCs injected into the gastrocnemius muscle showed lower percentages of CD4^+^ and CD8^+^ T cells compared to non-conditioned MuSCs, suggesting improved immune tolerance. Furthermore, conditioning with IFN-γ increased expression of MHC-I and PD-L1, although MHC-II expression remained unchanged. Shen et al. found similar results in CCL4-mediated liver injury model, wherein MuSCs under IFN-γ influence showed increased PD-L1 expression (Shen et al., 2022). Furthermore, similar results were seen *in vitro* when human atrial appendage-derived cells were cultured in the presence of IFN-γ and found to have increased expression of PD-L1 with lower expression of proinflammatory molecules and decreased immunogenicity (Diedrichs et al., 2019). Finally, our LacZ stains showed improved MuSC viability following treatment with hypoxia alone vs. hypoxia with a PD-L1 inhibitor provided evidence that hypoxia induction of PD-L1 expression, at least in part, is responsible improved survival of MuSCs grafts. Taken in combination with our data showing decreased immune infiltration, these findings underscore the complexity of the immune response to transplanted cells, and the potential for conditioning strategies, such as hypoxia and IFN-γ, to enhance transplant survival and efficacy.

## Conclusions/future directions

Given the limited availability of therapies for muscular dystrophy, our study offers a novel solution to a persistent challenge in cellular therapy: immunorejection. This study demonstrates that hypoxic induction of PD-L1 can condition muscle stem cells to enhance transplant retention and reduce inflammatory response. Additionally, our findings provide further evidence of hypoxia conditioning, with or without IFN-γ, significantly increased PD-L1 surface expression and highlights the crucial role of PD-L1 upregulation in reducing T cell immune responses. Overall, our results suggest that conditioning with stressors such as hypoxia or inflammatory cytokines like IFN-γ prior to cell transplantation therapies leads to improved immune tolerance and survival once grafted into a host. Prior to adapting MuSC into clinical therapies, future experiments utilizing *in vitro*, and *in vivo* conditions are needed to further understand the mechanisms of the immune privilege response across innate and adaptive immune cell types to justify the immune modulating effects of hypoxia and IFN-γ conditioning prior to cell transplantation. These experiments can focus on exploring cell viability and survival, particularly at time points leading into weeks and months after stem cell implantation. Additional studies can be done to include measurements of muscle tissue function by staining and measuring dystrophin in MDX mice. Furthermore, functional studies can be done to measure changes in muscle fiber unit function after transplantation with conditioned muscle stem cells, for instance, the force contraction of the gastrocnemius muscle or other alterations to the muscle fibers that occur after cell transplantation.

## Data Availability

The datasets presented in this study can be found in online repositories. The names of the repository/repositories and accession number(s) can be found in the article/Supplementary Material.

## Supplementary material

SUPPLEMENTARY FIGURE S1
Stained whole membrane blots of **(A)** PD-L1 (ab213480) and **(B)** β-actin (NB600-501) from LacZ+ MuSC treated with normoxia (CTL) 24 h, 48 h, or 72 h 5% hypoxia represented from Figure 1B as n = 1 per treatment.

SUPPLEMENTARY FIGURE S2

**(A)** Stained whole membrane blot of β-actin (NB600-501) with PD-L1 (ab213480) represented from n = 3 LacZ+ MuSC represented from Figure 1C that were treated for 6 h with normoxia (CTL) or 5% hypoxia (HYP). **(B)** Stained whole membrane blot of β-actin with PD-L1 represented from n = 3 LacZ+ MuSC represented from Figure 1C that were treated for 12 h with normoxia (CTL) or 5% hypoxia (HYP).

SUPPLEMENTARY FIGURE S3
Gating strategies used to measure the surface markers of LacZ+ MuSCs after 72 h treatments with normoxia, hypoxia, normoxia + IFN‐γ, and hypoxia + IFN‐γ from Figure 1D.

SUPPLEMENTARY FIGURE S4

**(A)** Whole membrane blots of β-actin stained (NB600-501) and PD-L1 stained (ab213480) represented from Figure 2, n = 1 samples. **(B)** Whole membrane blots of β-actin stained and PD-L1 stained are represented in Figure 2, n = 2 and n = 3 samples.

SUPPLEMENTARY FIGURE S5

**(A–B)** Gating strategy after Flow cytometry analysis to measure the percentage of CD4+ (APC) and CD8+ (FITC) lymphocyte populations subtracted from the unstained controls from Figure 3A,B.

SUPPLEMENTARY FIGURE S6

**(A)** Whole membrane blots of β-actin stained (NB600-501) and PD-L1 stained (ab213480) represented from normoxia (CTL) Figure 4B, n = 3 samples. **(B)** Whole membrane blots of β-actin stained and PD-L1 stained represented from hypoxia (HYP) Figure 4B, n = 3 samples.

SUPPLEMENTARY FIGURE S7
Gating strategies used to measure *in vivo* CD4+ and CD8+ cells present in GA muscle of mice 3d post 72 h, 5% hypoxia or hypoxia + IFN‐γ treatment on LacZ+ MuSCs from Figure 4D.

## Glossary

APC: Allophycocyanin
CD34: Cluster of Differentiation 34
CD4: Cluster of Differentiation 4
CD45: Cluster of Differentiation 45
CD8: Cluster of Differentiation 8
cDNA: Complementary Deoxyribonucleic Acid
**CO**_2_: Carbon Dioxide
DMD: Duchenne Muscular Dystrophy
DMEM: Dulbecco’s Modified Eagle Medium
DMSO: Dimethyl sulfoxide
FBS: Fetal Bovine Serum
FITC: Fluorescein Isothiocyanate
FSC-A: Forward Scatter Area
FSCH: Forward Scatter Height
GM: Gastrocnemius Muscle
**H**_**2**_ **O**_**2**_: Hydrogen peroxide
HBSS: Hanks’ Balanced Salt Solution
HIF-1α: Hypoxia-Inducible Factor 1α
HIF-2α: Hypoxia-Inducible Factor 2α
HGF: Hepatocyte Growth Factor
HRP: Horseradish Peroxidase
HYP: Hypoxia
ICAM: Intercellular Adhesion Molecule 1
IFN-γ: Interferon-γ
IgG: Immunoglobulin G
IGF-1: Insulin-Like Growth Factor-1
IL-4: Interleukin-4
IL-8: Interleukin-8
Ki67: Antigen Kiel 67
MDSC: Muscle Derived Stem Cells
MHC I: Major Histocompatibility Complex I
MHC II: Major Histocompatibility Complex II
mRNA: Messenger Ribonucleic Acid
MTT: 3-(4,5-dimethylthiazol-2-yl)-2,5-diphenyltetrazolium bromide
MuSC: Muscle Stem Cells
Myf5: Myogenic Factor 5
MyHC7: Myosin Heavy Chain 7
MyoD: Myoblast Determination Protein 1
OD: Optical Density
Pax7: Paired Box 7
PD-L1: Programmed Death Ligand 1
PVDF: Polyvinylidene fluoride
qPCR: Quantitative Polymerase Chain Reaction
RNA: Ribonucleic Acid
Sca1: Stem Cell Antigen-1
SDS: Sodium Dodecyl Sulfate
SDF-1: Stromal Cell-Derived Factor 1
SSC-A: Side Scatter Area
SSC-H: Side Scatter Height
SSC-W: Side Scatter Pulse Width
ssRNA: Single-Stranded Ribonucleic Acid
STDV: Standard Deviation
TLR: Toll-Like Receptor
VEGF: Vascular Endothelial Growth Factor
