# Supplementary figures and images for: Hypoxia-induced PD-L1 expression and modulation of muscle stem cell allograft rejection
Source: Front Pharmacol. 2024 Nov 1;15:1471563. doi: 10.3389/fphar.2024.1471563 (PMC11564730; doi:10.3389/fphar.2024.1471563)

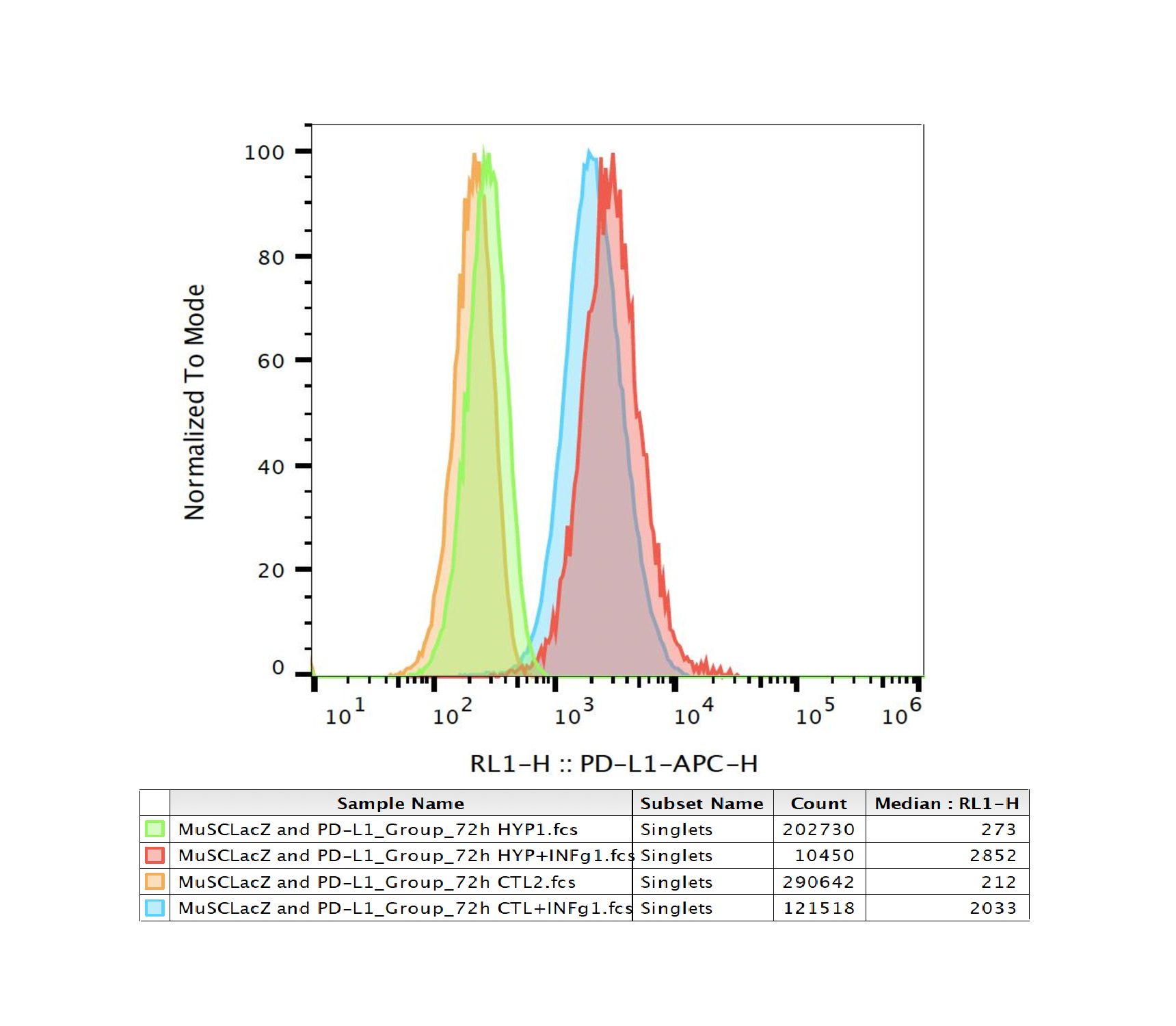

Supplement: Supplementary file 1 [file Image3.JPEG]

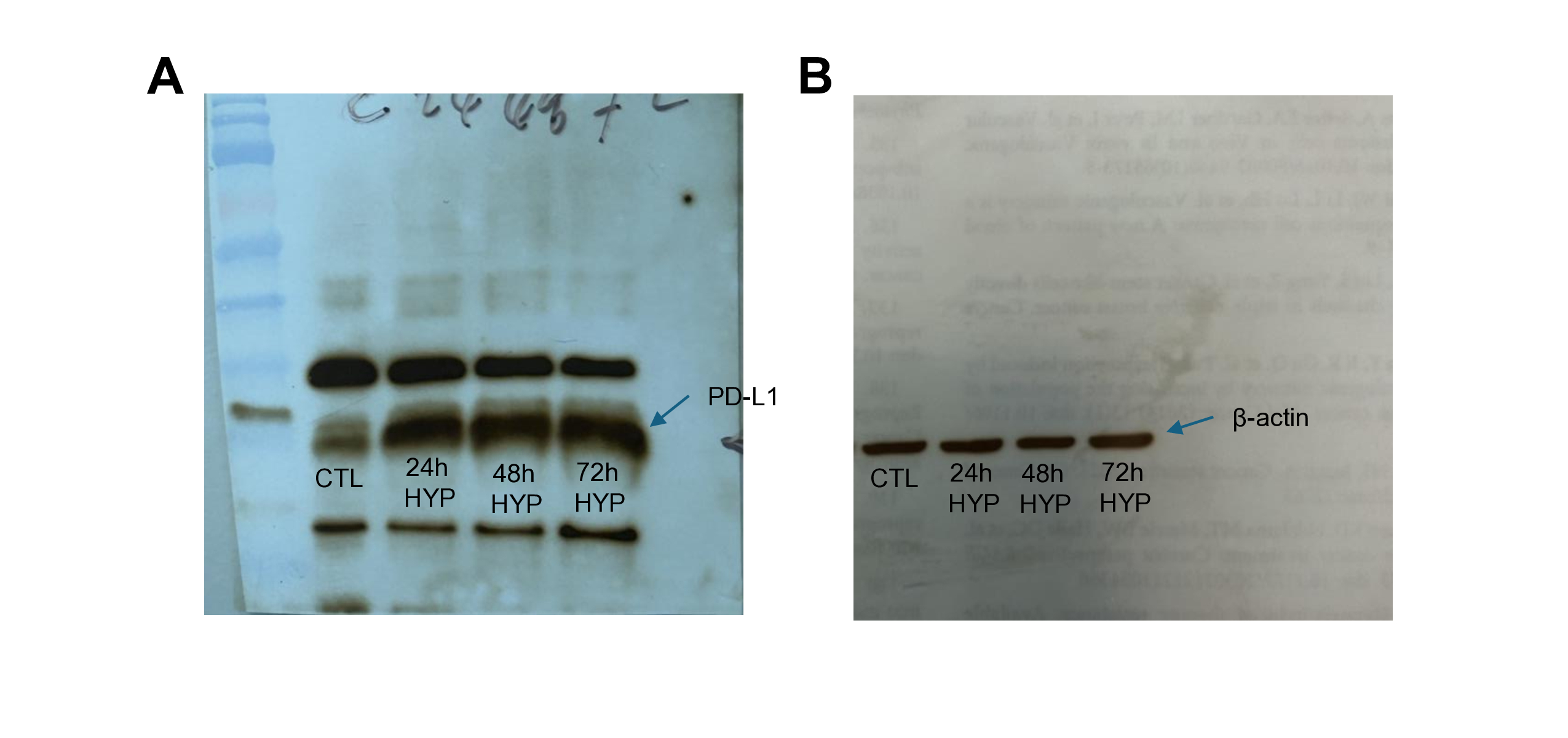

Supplement: Supplementary file 3 [file Image1.JPEG]

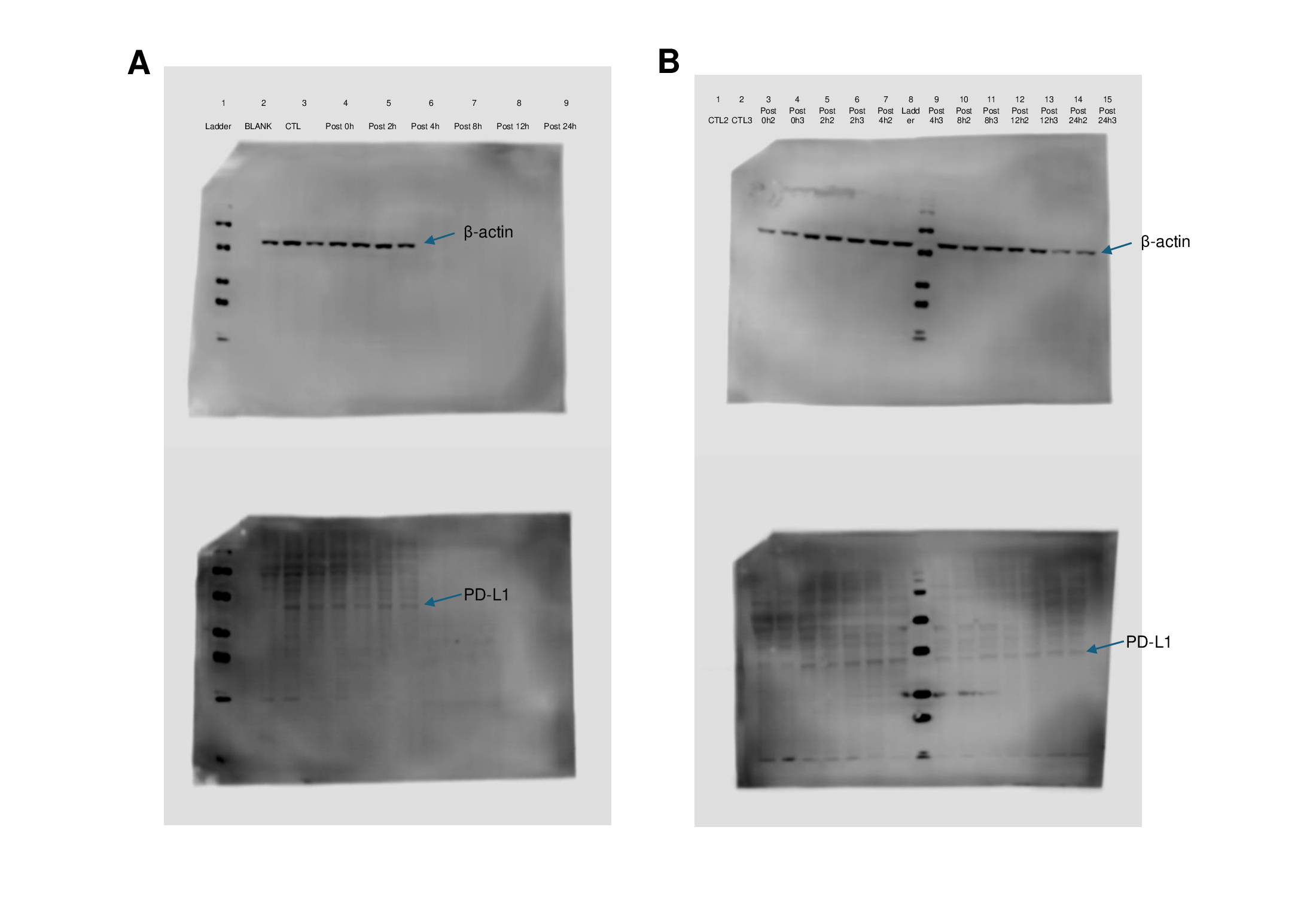

Supplement: Supplementary file 4 [file Image4.JPEG]

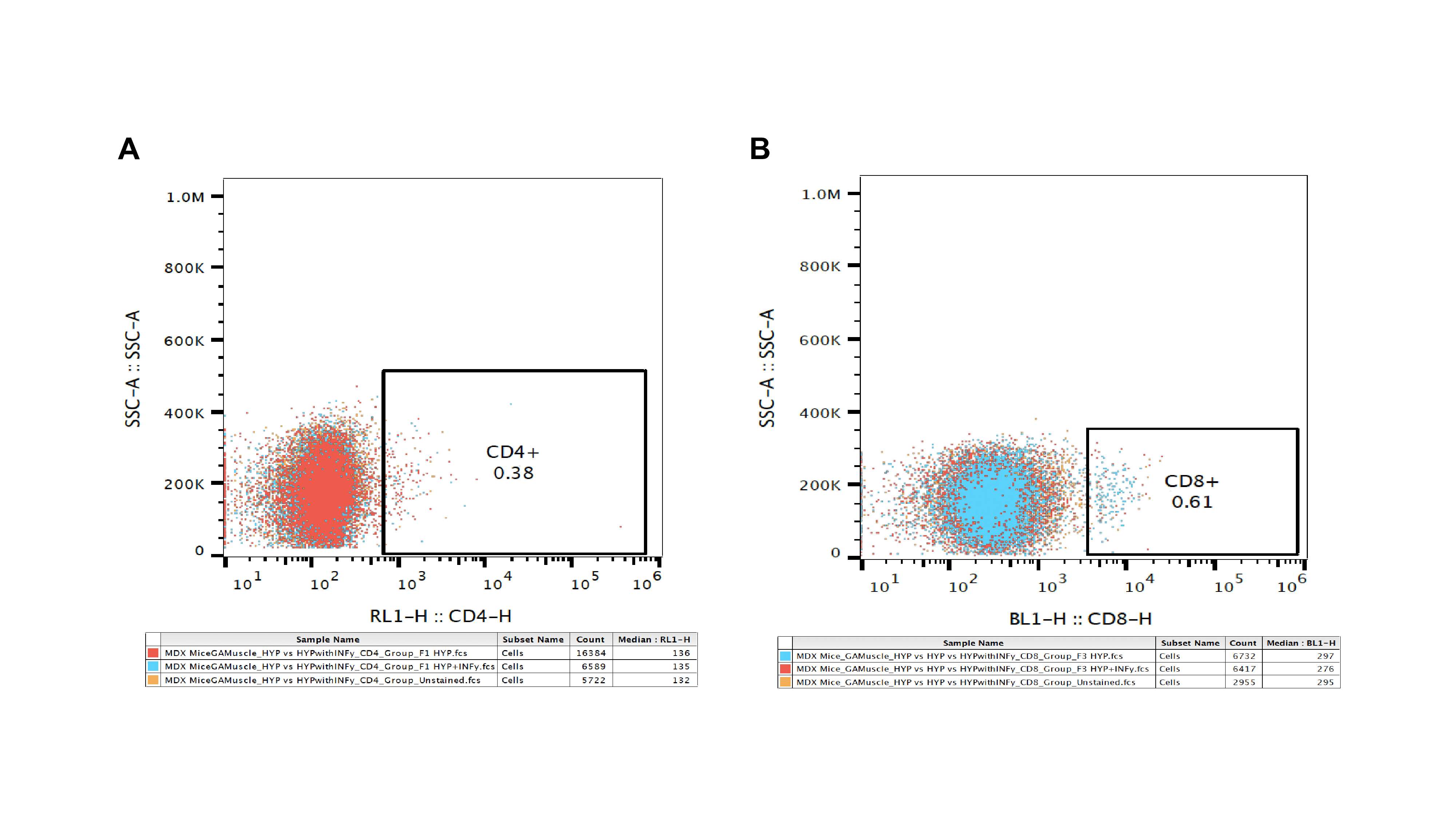

Supplement: Supplementary file 5 [file Image7.JPEG]

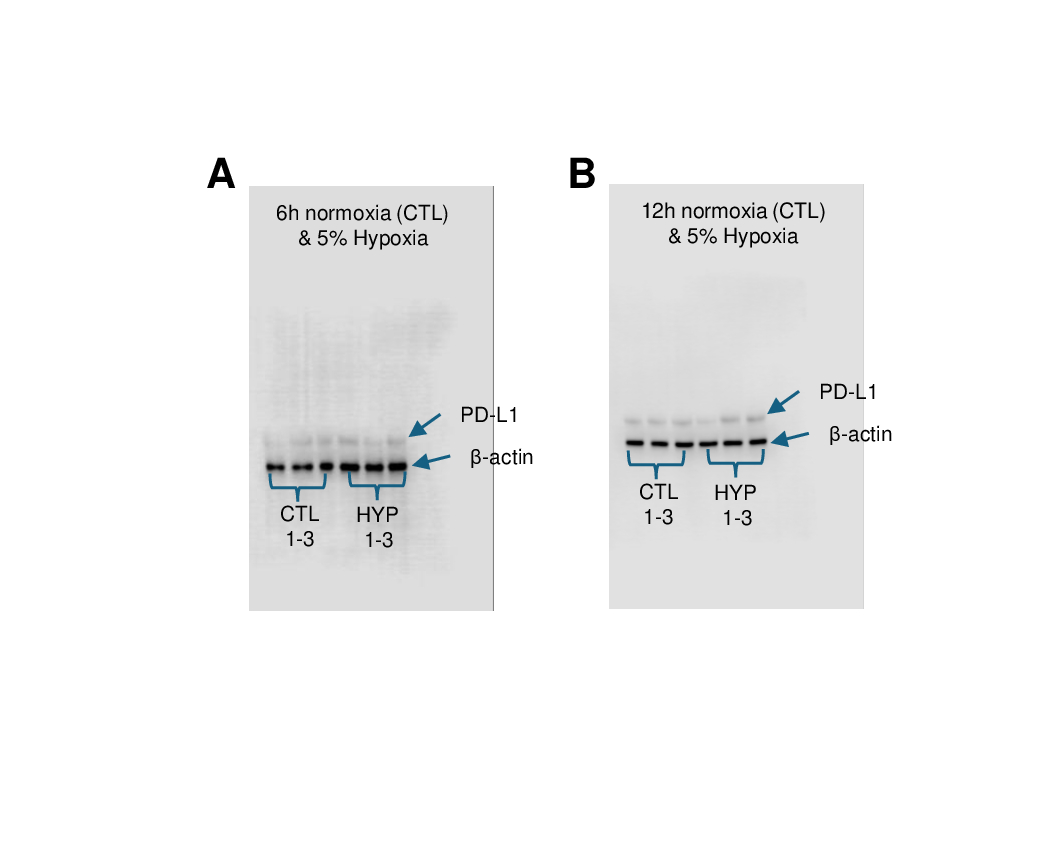

Supplement: Supplementary file 6 [file Image2.JPEG]

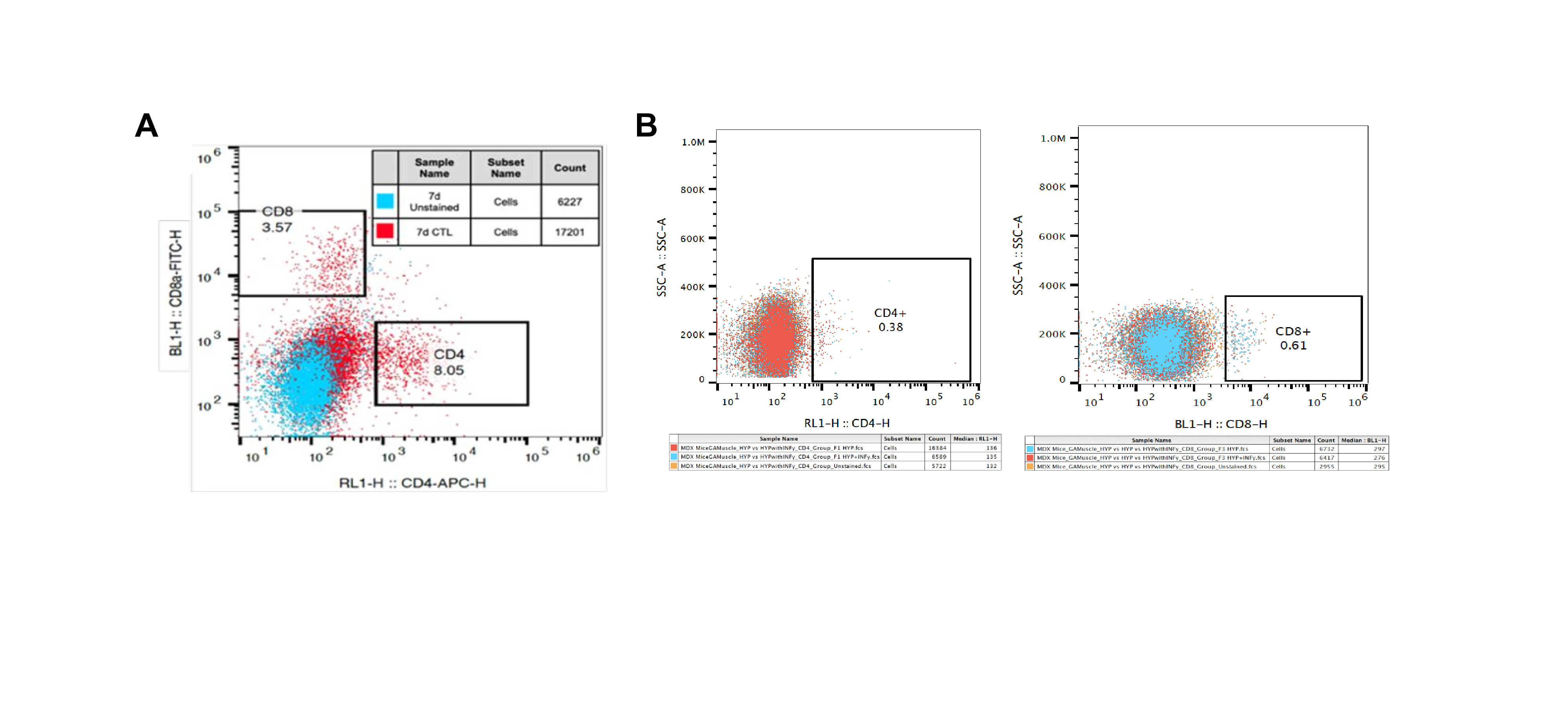

Supplement: Supplementary file 7 [file Image5.JPEG]

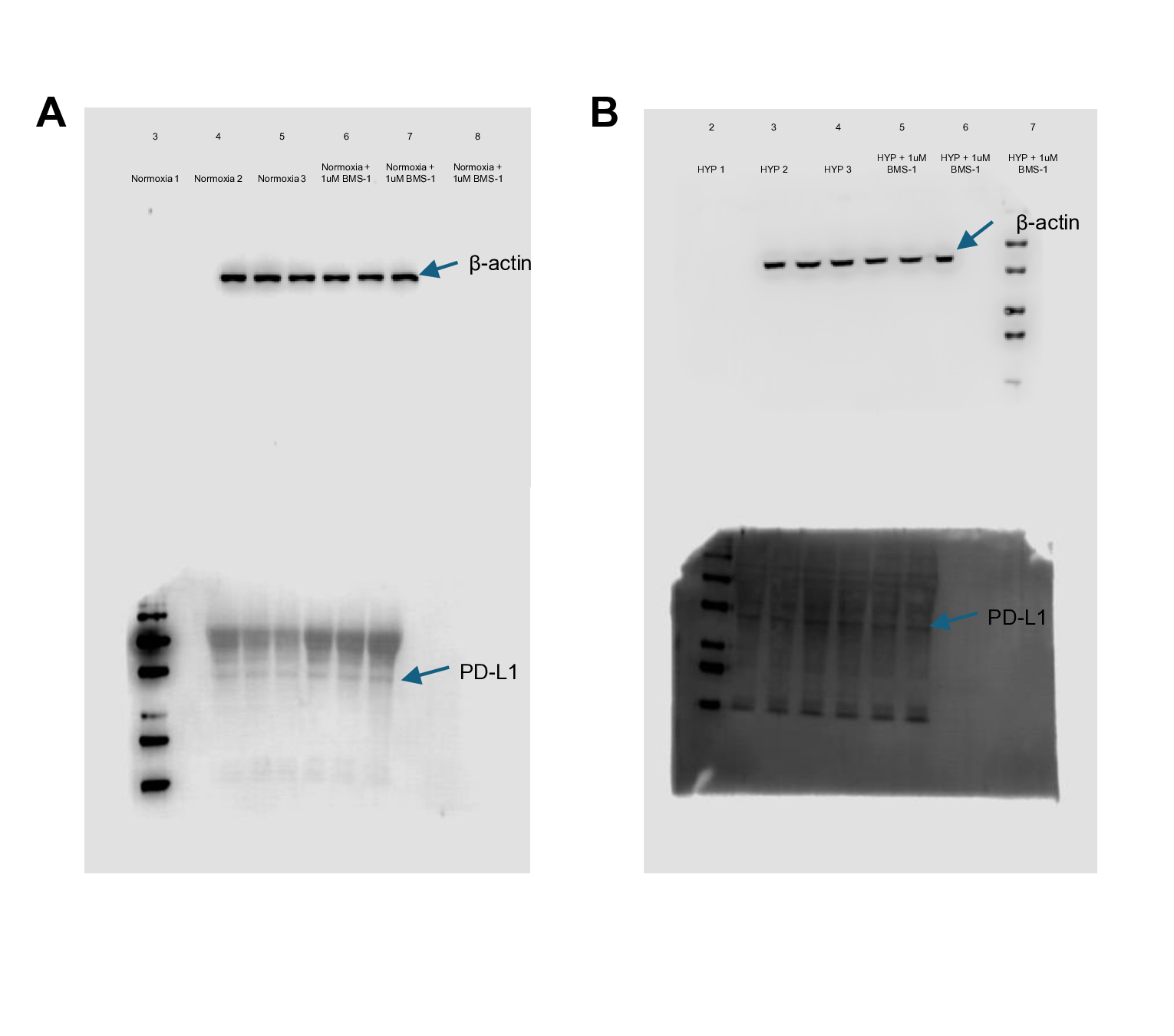

Supplement: Supplementary file 8 [file Image6.JPEG]
