# Supplementary material for: Hypoxia-induced PD-L1 expression and modulation of muscle stem cell allograft rejection
Source: Front Pharmacol. 2024 Nov 1;15:1471563. doi: 10.3389/fphar.2024.1471563 (PMC11564730; doi:10.3389/fphar.2024.1471563)
Supplement: Supplementary file 2 [file Table1.pdf]

Table 1: Primers Used for LacZ+ MuSC Characterization

Gene	Primer Probe	Sequence
CD45	Sense	5'ACTTCTGGCCTTTGGATTTGC'3
	Antisense	5'TGTGCTTGGAGGGTCAGTG'3
CD34	Sense	5'ATGGCGCTGGGTAGCTCTCT'3
	Antisense	5'GGCTGGTGTGGTCTTACTGCTG'3
Sca1	Sense	5'AGGAGGCAGCAGTTATTGTGG'3
	Antisense	5'CGTTGACCTTAGTACCCAGGA'3
Pax7	Sense	5'TCTCCAAGATTCTGTGCCGAT'3
	Antisense	5'CGGGGTTCTCTCTCTTATACTCC'3
Myf5	Sense	5'ACAGCAGCTTTGACAGCATC'3
	Antisense	5'AAGCAATCCAAGCTGGACAC'3
MyoD	Sense	5' CCACTCCGGGACATAGACTTG'3
	Antisense	5'AAAAGCGCAGGTCTGGTGAG'3
Myogenin	Sense	5'GGTGTGTAAGAGGAAGTCTGT'3
	Antisense	5'TAGGCGCTCAATGTACTGGAT'3
MyHC7	Sense	5'CTCAAGCTGCTCAGCAATCTATTT'3
	Antisense	5' GGAGCGCAAGTTTGTCATAAGT'3
Ki67	Sense	5'GCAGGTTAGCACTGTTATGAAAAC'3
	Antisense	5'GGGCCTTGGCTGTTTTACATT'3
β-actin	Sense	5'GGCTGTATTCCCCTCCATCG'3
	Antisense	5'CCAGTTGGTAACAATGCCATG'3
